# Effects of single-point acupuncture (HT7) in the prevention of test anxiety: Results of a RCT

**DOI:** 10.1371/journal.pone.0202659

**Published:** 2018-08-30

**Authors:** Johannes Fleckenstein, Peter Krüger, Karl-Peter Ittner

**Affiliations:** 1 Department of TCM/Acupuncture, Institute of Complementary Medicine, University of Bern, Personalhaus 4 Inselspital, Bern, Switzerland; 2 University Hospital Regensburg, Franz-Josef-Strauß-Allee 11, Regensburg, Germany; 3 Department of Sports Medicine, Institute of Sports Sciences, Goethe-University Frankfurt, Frankfurt am Main, Germany; Brown University, UNITED STATES

## Abstract

**Background:**

The number of students using neuro enhancement to improve their performance and to prevent test anxiety is increasing. The acupuncture point Heart 7 (HT7) has been described as being prominent in reducing states of anxiety.

**Methods:**

We conducted a randomized placebo-controlled, two-armed pilot trial to investigate the efficacy of a single-point acupuncture treatment at bilateral HT7 compared to sham laser acupuncture on test anxiety. Test anxiety was induced applying the standardised protocol of the Trier Social Stress Test. Outcome measures included saliva samples analysed for cortisol and amylase, anxiety questionnaires and heart rate variability.

**Results:**

Twenty-five male subjects (age 28 ± 5 years) were allocated to either verum acupuncture (n = 12) or sham laser acupuncture (n = 13). Cortisol peaked 20 min after the stress test (2-fold, 18.11 ± 2 nmol/l) and amylase 10 min after (2-fold, 259 ± 49 U/ml) with no difference between groups. There were no differences between groups regarding either anxiety questionnaires or physiological parameters. Compared to reference data (3-fold increase in cortisol), increase in stress hormones and heart rate seemed somewhat reduced.

**Conclusions:**

Acupuncture may be a possible approach for the treatment of anxiety. Due to the lack of a no control treatment group, we cannot determine the magnitude of possible specific needle effects at HT7 to promote specific effects in the neuroendocrine system. Finally this study only examines the efficacy of a single time treatment.

## Introduction

The German Federal Ministry of Education and Research reports that 13% of all first-year undergraduate students seek counselling services for test anxiety [1]. Test anxiety also seems to be more prevalent in students who dropped their first study major and started another. In this group, 17% sought counselling services. It was also shown that the number of students affected by test anxiety and require help rises with the age of the student [1].

The experience of anxiety in a test situation has several different components. There is an affective component, which occurs as an unpleasurable, nervous feeling of affective excitement, a cognitive component, which includes concern for impending failure and its possible consequences, a physiological component, for example an increased heart rate, sweating or nausea, and a motivational component, which involves escape and avoidance tendencies [2]. In this regard, current investigations distinguish between situationally experienced test anxiety, or so-called "state test anxiety," and habitual personality-specific test anxiety, which is referred to as "trait test anxiety" [3].

The consequence of such anxiety is that the performance of complex and difficult tasks, which require attention, decreases. The effect on motivation is ambivalent. Test anxiety is known to reduce interest and motivation but can be beneficial as well since students are more focused on avoiding errors. In general, however, it has been proven to significantly weaken one's abilities to solve cognitively challenging tasks [3].

Middendorf et al [4] showed in their survey that the most popular options for students to relieve anxiety and stress is meeting with friends (69%) and entertainment (67%). Other remedies are sleeping (63%), sports activities (58%) or relaxing and wellness (46%).Drinking coffee is a common strategy for staying awake and maintaining concentration. Diekelmann et al. investigated whether caffeine has any effect on memory after sleep deprivation. The group given caffeine had 10% less false memories than those who did not receive caffeine [5]. The number of students using neuro enhancement to improve their performance and to prevent test anxiety is ever increasing. A US survey estimated that nearly 7% of students in US universities had used prescription stimulants against anxiety, and that on some campuses up to 25% of students had used them in the past year [6]. Twelve percent of all students questioned in 2012 had used one or more substances to cope with the requirements of studying since starting their studies [4]. Five percent of these students reported having taken prescription drugs, including analgetics, sedatives, psychostimulants or stimulants, and another 5% so-called soft-enhancers.

Previous studies have shown that acupuncture can be a beneficial, non-pharmacological alternative for the reduction of test anxiety [7, 8], and the acupuncture point Heart 7 has always been a central part of the point concepts used in these studies. A variety of animal studies investigated the effects of HT-7 on anxiety in drug and alcohol withdrawal. Suggested mechanism to attenuate anxiety-like behaviour include the activation of the dopaminergic system [9] and through the mediation of the GABA-A receptor system [10]. Clinical evidence favouring these effects on anxiety is still limited. An uncontrolled pilot study claimed HT 7 to cause point-specific effects [11]. A recent RCT showed acupressure of HT7 in combination with LI4 to be superior to sham and control in relieving anxiety [12].

A further limitation in these previous studies is the lack of a standardized setting to provoke anxiety. Kirschbaum et al. addressed this problem by designing a protocol known as the Trier Social Stress Test, a scientifically validated instrument for the provocation of psychobiological stress [13].

This study aimed to reproduce the described effects of acupuncture at HT7 on anxiety in a standardised manner.

## Methods

We conducted a randomized, controlled, two-armed pilot trial from March to April 2014 to investigate the efficacy of a single acupuncture treatment at bilateral acupuncture point Heart 7 on experimental acute test anxiety as compared to sham laser acupuncture. The study was performed according to the guidelines stated in the Declaration of Helsinki (Version Fortaleza 2012), and ethical approval was granted by the Ethics Committee of the University of Regensburg, Germany. The ClinicalTrials.gov identifier is NCT02142231 (see S1, S2 and S3 Files).

We screened male students of the Medical School (3^rd^ to 5^th^ year) at the University of Regensburg for eligibility. Written informed consent was obtained from all participants. Inclusion criteria were smoking cessation for 24 hours, compliance and a history of test anxiety with a value > 4 cm on a visual analogue scale (VAS, range 0–10 cm). Exclusion criteria were severe physical or psychological illness, a psychiatric record in the medical history, the continuous intake of psychiatric medication, tranquilizers or neuro-enhancers, an acupuncture treatment within the last 4 weeks, current hang-over, drug consumption, or smoking habits with more than 5 cigarettes per day.

An independent person prepared 25 sealed, opaque envelopes on the basis of a randomization sequence generated by a computer-based algorithm (Research Randomizer, Version 4.0) in order to allocate 12 participants into each group. Participants were allocated by means of the ascending numbered envelopes to one of the two trial arms receiving either single needle acupuncture at point Heart 7 (ACU) or laser acupuncture (LAS).

Participants laid comfortably on a bed while acupuncture needles (Seirin^®^ 0.15 mm diameter and 15 mm length) were placed bilaterally into the acupuncture point Heart 7 (ACU) until the sensation of deqi was achieved. No additional manipulation was sought. Needle-in time was 20 minutes. The practitioner had achieved the A-level Grade of the German Medical Acupuncture Association.

Sham laser acupuncture was performed at the same acupuncture point, bilaterally, without palpating or touching the skin (laser pen manufactured by 3B Scientific,GmbH, Hamburg, Germany). Treatment was one minute per point with an additional 18 minutes of resting time. Both patient and therapist were blinded to the fact that a sham laser was used instead of a verum laser, as published previously.[14]

The experimental sessions were conducted between 13:00 and 18:00 o’clock. Participants were told to refrain from eating and drinking anything but water for 2 h, and from intense physical activity, caffeine, nicotine, and alcohol for 24 h before the experiment. The ECG recording equipment (Polar) was attached first and then the recording was started.

To induce test anxiety, the Trier Social Stress Test (TSST) was used, combining a 10 min preparation phase followed by a 5 min mock job interview, and a 5 min mental arithmetic exercise [13]. Both tasks were performed 2 m in front of two evaluative panel members (not part of the study team), dressed in white laboratory coats, and a conspicuous video camera and microphone. The socio-evaluative character of this performance task was further underscored by presenting the panel members (a retired male finance manager and a female psychologist) as experts in evaluation of nonverbal behaviour. The TSST reliably activates both the HPA-axis and the sympathetic nervous system. During recovery, subjects remained seated in a quiet room for 60 min.

The main outcome was the assessment of cortisol in the saliva (8 different assessment times). All saliva probes were sampled by means of the standardized Salivetten^®^ Cups, (Sarstedt, Nürmbrecht, Germany). Participants chew for 30 seconds on an absorptive tissue and subsequently place saliva in the cup. The cups are then closed and stored at -20°C until further analysis. All laboratory analyses were performed by DRESDEN LAB SERVICE GmbH, Dresden, Germany.

Secondary outcomes included the assessment of amylase in the saliva. The following questionnaires were used to assess different dimensions of stress and anxiety: the Multidimensional Mood Questionnaire MDBF [15], the Primary Appraisal Secondary Appraisal scale PASA [16], the State-Trait Anxiety Inventory STAI [17], and perceived stress on a 10 cm visual analogue scale (VAS; with 0 = no stress and 10 = worst imaginary stress). Times of assessment adhered to the TSST protocol as published previously [13].

A Polar RS800 device (Polar Electro GmbH Germany, D- 64572 Büttelborn) was used to constantly record a high resolution ECG during the experiment. We extracted the heart rate (HR) and the heart rate variability (HRV) from the raw data.

Expectations about the outcome are the main modifying variables of the placebo effect. Participants were therefore asked to evaluate whether their satisfaction and expectations were met by means of a questionnaire as suggested by Vincent and Lewith [18]. This questionnaire is comprised of 4 items that are evaluated on a 10 cm VAS: (1) Alleviation, (2) Recommendation, (3) Logic, and 4 Other.

A power analysis was performed using G*Power (University of Düsseldorf, Germany) estimating a small to medium effect size (d = 0.29, α-error = 0.05, power = 0.95)) and a time-dependent progression, according to a suggested total sample size of 24 subjects (i.e. 12 per group).

All data was distributed in a way that is consistent with normality, and are thus expressed as mean ± standard deviation.

To analyse longitudinal data (more than 2 time points; changes to baseline), we applied a mixed-effects analysis, i.e. a time x group model (ANOVA for repeated measures). The model was applied to analyse the effects on cortisol, amylase, heart rate and heart rate variability. Data was analysed according to Mauchly’s test for sphericity and the Greenhouse-Geisser correction was used in case sphericity was not present. Weight and trait anxiety at baseline were included as covariates. If statistically significant, the respective outcome variable was followed by post-hoc pairwise comparisons (unpaired t-test) of change scores between each of the time points and baseline.

All other data was analysed applying unpaired Student’s t-tests for inter-group, or paired t-tests for intra-group analysis.

## Results

**Fig 1:**
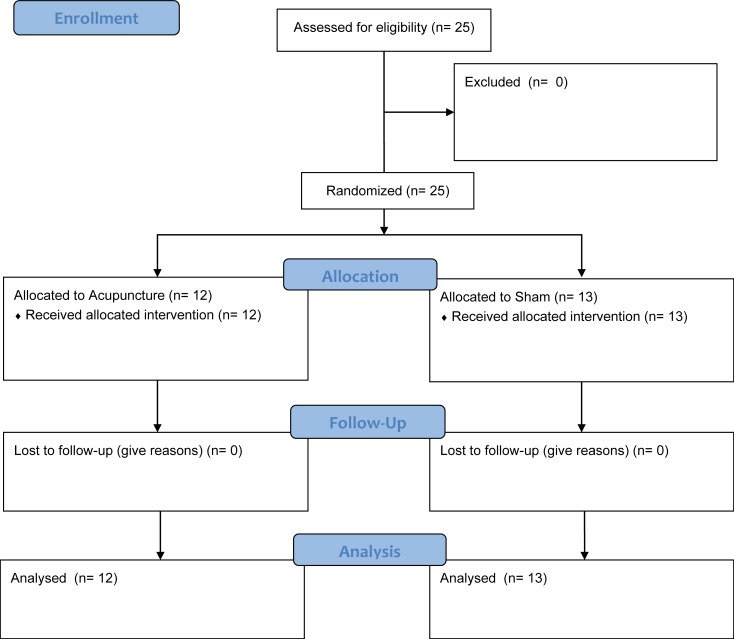
Consort study flow chart.

Twenty-five male volunteers (mean age 27.9 (SD 4.6) years, height 182.0 (6.5) cm, weight 79.7 (10.1) kg) participated in this study, with no drop-outs (Fig 1). Global test anxiety was reported with 7.4 (1.3) cm VAS (range 6.0–10.0) at baseline. Volunteers were randomized into two groups with 12 subjects allocated to needle acupuncture and 13 subjects to sham laser acupuncture. Groups differed in weight and in regard to their state of anxiety (STAI-T, see Table 1). As both variables are known to influence each other [19], they were used as covariates in the analysis of the main effects.

**Table 1:** Participants’ characteristics. Data is expressed as means (SD), with paired Students’ t-test indicating statistical differences between the Sham and the Verum Group. 95% CI = confidence interval, STAI state trait anxiety inventory (with the subscores–T trait and–S state), MDBF multidimensional mood questionnaire, PASA primary appraisal secondary appraisal, HR heart rate HRV heart rate variability.

	Verum Acupuncture	Sham Laser	Students t-test
	(n = 12)	(n = 13)	p-value (95% CI)
**Age (yrs)**	28.1 (5.5)	27.7 (3.7)	0.836 (-3.3;4.2)
**Height (cm)**	182.8 (5.1)	181.2 (7.7)	0.549 (-3.8;7.1)
**Weight (kg)**	84 (10.2)	75.7 (8.5)	**0.036** (0.6;16.1)
**Test Anxiety_baseline_ cm VAS**	7.7 (1.4)	7.2 (1.2)	0.323 (-0.5;1.6)
**Perceived Stress in the Experiment (cm VAS)**	5.2 (1.7)	5.3 (1.5)	0.943 (-1.5;1.4)
**Comparability to Medical State Exam (cm VAS)**	3.9 (2.2)	4.1 (2.0)	0.806 (-2.1;1.7)
**Cortisol_baseline_ (nmol/l)**	7.7 (3.3)	11 (7.4)	0.158 (-8.1;1.5)
**Amylase_baseline_ (U/ml)**	103.4 (86.8)	138.3 (119.8)	0.417 (-122.1;52.3)
**Cortisol_peak (T4)_ (nmol/l)**	16.5 (10.5)	19.6 (9.5)	0.451 (-11.4;5.2)
**Amylase_peak (T3)_ (U/ml)**	235.5 (225)	280.7 (264.7)	0.652 (-249.3;159.0)
**STAI-T_baseline_**	33.0 (4.8)	38.8 (6.7)	**0.023** (-10.7;-0.88)
**MDBF_baseline_**	33.6 (2.8)	31.2 (9.7)	0.426 (-3.6;8.4)
**MDBF_post intervention_**	34.3 (2.5)	31.1 (9.6)	0.279 (-2.8;9.1)
**MDBF_post TSST_**	31.3 (3.5)	31.4 (3)	0.969 (-2.7;2.6)
**STAI-S_baseline_**	38 (5.6)	41 (11)	0.406 (-10.3;4.3)
**STAI-S_post intervention_**	32.3 (5.4)	35.8 (8.7)	0.253 (-9.5;2.6)
**STAI-S_post TSST_**	39.9 (7,9)	43.6 (7.4)	0.238 (-10.0;2.6)
**PASA_post TSST_**	-5.4 (5.6)	-3.8 (5.8)	0.479 (-6.4;3.1)
**HR_baseline_ (min^-1^)**	75.8 (12.4)	77.4 (17.7)	0.808 (-14.7;11.6)
**HR_post intervention_ (min^-1^)**	65.7 (11.2)	67.9 (14.2)	0.692 (-12.1;8.9)
**HR_TSST_ (min^-1^)**	87.4 (13.9)	91.2 (21.6)	0.619 (-19.5;11.9)
**HRV_baseline_ (ms)**	757.1 (270.2)	831.3 (197.6)	0.439 (-268.9;120.5)
**HRV_post intervention_ (ms)**	945.4 (163.1)	929.1 (189.7)	0.825 (-135.0;167.6)
**HRV_TSST_ (ms)**	708 (113.6)	705.4 (184.3)	0.969 (-130.2;135.2)

The perceived stressfulness of the TSST was rated as 5.3 (1.6) cm VAS (range 3.0–8.0) and the comparability to a medical state exam was valued as 4.0 (2.1) cm VAS (range 1.0–8.0) with no differences observed between groups.

Stress hormones in the sputum at baseline were cortisol = 9.4 (5.9) nmol/l and amylase = 121.6 (104.6) U/ml. The largest magnitude of cortisol was Δ8.7 (11.8) nmol/l at T4 (20 minutes after the stress test; p = 0.001) and of Δ137.4 (161.6) U/ml at T3 (10 minutes after the stress test; p < 0.001).

**Fig 2:**
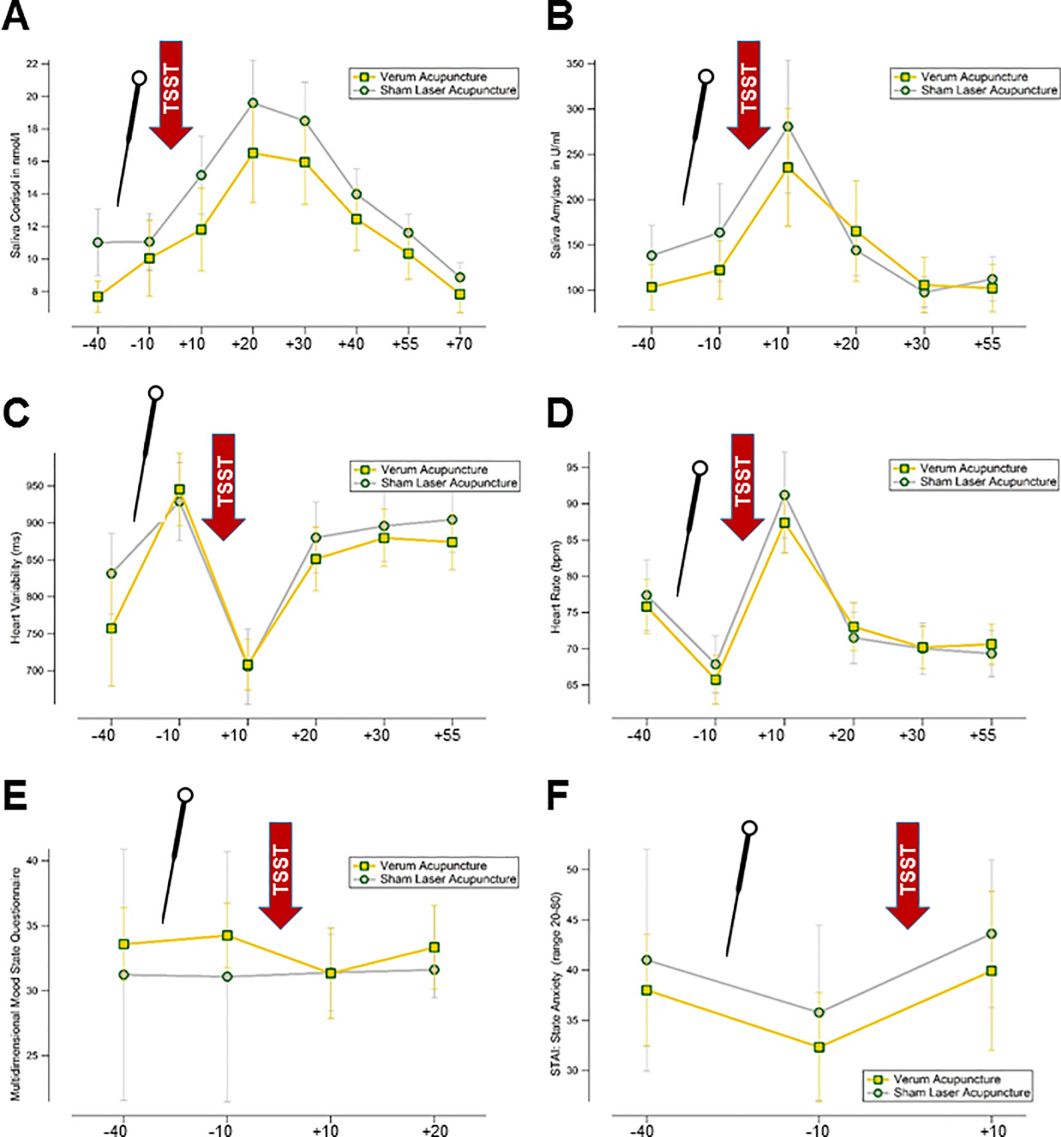
Describes the physiological and psychological effects of acupuncture on test anxiety, i.e. the time-course of (A) saliva cortisol and (B) saliva amylase. (C) and (D) depict the ECG analysis with respect to heart rate and heart rate variability. (D) is the score of the MDMF and (F) the scores of the STAI-state anxiety. The red flashes labelled TSST indicate the occurrence of the stress test, the acupuncture needle depicts the time of intervention (30 minutes prior to the TSST). The times are as follows: -40 and -10 minutes previous to the TSST, and +10, +20, +30, +40, +55, +70 minutes thereafter (as suggested by Kirschbaum et al., 1993).

Time x group analysis of changes to baseline revealed no significant differences in the increase of cortisol (7x2; F(2;12) = 0.5 p = 0.811) or amylase (5x2; F(3;8) = 1.2 p = 0.325) between groups (see Fig 2A and 2B).

Time x group analysis of changes to baseline revealed no significant differences between groups with respect to other psychological outcomes as follows: MDBF (3x2; F(2;4) = 1.4 p = 0.257; Fig 2E), and STAI-S (2x2; F(2;2) = 0.08 p = 0.927; Fig 2F). Changes in PASA did not differ between groups (unpaired t-test p = 0.524 (95%-CI -2.8;5.3))

Time x group analysis of changes to baseline revealed no significant differences with respect to the perceived stress (on a VAS scale) during the experiment (4x2; F(2;6) = 0.4 p = 0.832).

The heart rate (at baseline 76.7 [15.2] min^-1^) significantly decreased after intervention/resting to 66.9 (12.7) min^-1^ (p < 0.001), and peaked during the TSST at 89.5 (18.2) min^-1^ (p < 0.001). The heart rate variability (at baseline was 795.7 [233.3] ms) significantly increased after intervention/resting to 936.6 (174.4) ms (p < 0.001), and was shortened during the TSST (706.6 [152.8] ms, p < 0.001).

Time x group (5x2) analysis of changes to baseline revealed no significant differences between groups with respect to heart rate (F(2;8) = 0.6 p = 0.630) or heart rate variability ((F(2;8) = 0.9 p = 0.499; Fig 2C and 2D).

We performed a credibility assessment according to Vincent, and observed no differences with respect to the four variables at baseline, or at the end of the study (Table 2).

**Table 2:** Credibility assessment. The assessment was perfomed at baseline and at the end of the study indicating no significant differences in regard to expectations between the Sham and the Verum Group. 95% CI = confidence interval.

	Verum Acupuncture	Sham Laser	Students t-test (95% CI)
**Alleviation_baseline_**	4.69 (2.67)	3.35 (2.35)	0.194 (-0.7;3.4)
**Recommendation_baseline_**	5.68 (2.11)	4.23 (2.56)	0.137 (-0.5;3.4)
**Logic_baseline_**	4.48 (2.65)	3.6 (2.04)	0.358 (-1.1;2.8)
**Other_baseline_**	7.15 (1.28)	5.91 (2.44)	0.130 (-0.4;2.9)
**Alleviation_end of study_**	5.38 (2.38)	3.43 (2.54)	0.061 (-0.1;4.0)
**Recommendation_end of study_**	5.71 (2.16)	4.91 (2.62)	0.415 (-1.2;2.8)
**Logic_end of study_**	5.17 (2.24)	3.58 (2.56)	0.113 (-0.4;3.6)
**Other_end of study_**	7.33 (1.39)	5.58 (2.88)	0.065 (-0.1;3.6)

We could not detect any side effects.

## Discussion

This study demonstrated that treatment at the acupuncture point Heart 7 did not show any treatment-specific effects on the physiological response in a setting of standardized anxiety when compared to a sham control. However, when compared to reference scores [13], the increase in the peptides cortisol and amylase was 2-fold, which is less than expected (3 up to 4-fold). This may be due either to indirect effects (so called treatment-dependent unspecific effects [14]), or to individual differences between the study populations. The TSST paradigm has been studied in depth and is a validated test protocol to provoke anxiety [20]. Still it may not be equivalent to an acute stress response in real life. Our data suggest that care and attention given during a real or sham acupuncture treatment could already reduce the physiological and psychological stress response to some extent.

We believe that the methodology of this study adheres to the latest recommendations defined in the literature. We specifically avoided using a sham needle control due to the probable mixture of physiological stimuli when touching the skin [21]. Sham laser acupuncture is thought to be an accurate control for acupuncture since all psychological dimensions are addressed and physiological stimuli e.g. touching the skin can be avoided [14]. We also carefully adhered to the STRICTA and CONSORT criteria for conducting and reporting acupuncture trials (S1 and S2 Files; [22]). Furthermore, we only included male subjects, first due to the complex nature of the female endocrinology, and second because male subjects seem to be more vulnerable to psychosocial stress [23]. This improves on the one hand the homogeneity of the study, but on the other reduces its generalizability. Finally, we thoroughly followed the instructions described in the TSST handbook, as suggested by Kirschbaum et al. (1993), in order to ensure that our data is reliably comparable to other current or ongoing studies.

To our knowledge, there is no acupuncture study that has been performed using the TSST paradigm. Other studies in this field show that e.g. taiji [24], cognitive therapy [25], or psychotherapy all successfully reduced the stress response[26]. The increase in saliva cortisol seen in our study is no different than that obtained by the best treatment responders within these studies when compared to healthy controls. Thus, this strengthens the hypothesis that psychological factors are very powerful in reducing acute stress.

The hypothesis that acupuncture could be used to reduce stress is linked to the clinical observation that acupuncture treatments cause major relaxation in patients. This effect has been attributed to modulations in the autonomic nervous system [27]. In addition, acupuncture is also assumed to enhance parasympathetic stimulation [28].

Unfortunately, there are only a few studies investigating the impact of acupuncture on (test) anxiety. A recent study evaluating the effects of auricular acupuncture on the psychological dimensions of real exam anxiety in medical students barely shows any differences between verum and placebo treatment [29]. Auricular acupuncture for 4 weeks significantly decreased tension, anxiety, anger, and aggression in psychiatric patients, as assessed by the visual analogue scales [30]. An integrative review from 2016 found 19 articles with 11 of them generally supporting the use of acupuncture to reduce anxiety [31]. Nonetheless, this review article still has some risk of bias. Studies investigating the effects of acupuncture on both anxiety and cortisol are rare. One clinical study exists that showed that acupuncture does not significantly reduce anxiety and serum cortisol in women undergoing embryo transfer when compared to placebo [32]. In female dysphonic speakers, acupuncture did not reduce salivary cortisol or emotional stress when compared to sham [33]. A further study investigating the effects of acupuncture or attention in people suffering chronic stress observed a trend towards significance in improving the diurnal profile of salivary cortisol for both groups [32]. In athletes, acupuncture significantly increased salivary immunoglobulin A and reduce salivary cortisol prior to competition [34]. Acupuncture significantly reduced the extent of orthostatic stress correlates with the change in morning salivary cortisol [28]. Finally, acupuncture has been shown to decrease the serum cortisol concentration of horses subjected to startle tests, which are stressful conditions for the animals [35].

It is important to state that the above-mentioned studies did not all apply acupuncture at Heart 7, but included other points or systems as well (e.g. auricular acupuncture). In this study, we chose Heart 7 due to both its historic and scientific importance. Heart 7 (神門, pinyin: shen men, translation: spirit gate) is considered to calm both mood and spirit. Experimental research in rats suggests that this point inhibits cocaine-induced locomotor activity, and that its action is mediated by A-fibre activation of the ulnar nerve [36]. There is also evidence that stimulation of the specific acupoint Heart 7 (and not others) helps to normalize the release of dopamine in the mesolimbic system following ethanol withdrawal [37], thereby exerting anxiolytic effects [38]. Acupuncture at HT7 was shown to reduce anxiety-related behaviours and modulate the hypothalamic-pituitary-adrenal system in a maternal separation model [39]. Although we are aware that these are only small observations drawing a fragmentary sketch as to how acupuncture may contribute to the psychoneuroendocrine system, but considered together they provide some evidence that acupuncture at Heart 7 may reduce not only endocrine parameters of stress, but also perceived anxiety.

The major limitation of this study is the lack of a no treatment control, which would have been helpful to evaluate and rate the clinical impact of the obtained results. In addition, the sample size estimation is hypothetical as there is no clinical cut-off in increase indicating cortisol levels to represent less stress. Even as our data is comparable to reference data published by other groups, we are aware about the possible chance of bias when interpreting the results. The small sample size may also have caused a loss of effect in outcomes close to significance. This research is of a preliminary and hypothesis-generating nature.

## Conclusion

Acupuncture may be a possible approach for the treatment of anxiety. The data of our study suggest that treatment-related unspecific effects (e.g. attention) are part of this observation. Due to the lack of a no control treatment group, we cannot determine the magnitude of possible specific needle effects, showing the treatment at the traditional acupuncture point Heart 7 to promote specific effects in the neuroendocrine system. Finally this study only examines the efficacy of a single time treatment, future studies are necessary to show whether the specific effects of verum acupuncture are more sustainable in the long run and whether they may be of additional benefit.

## Supporting information

S1 File Consort 2010 checklist. Checklist according to the CONSORT criteria.
(PDF)

S2 File Stricta_checklist_in_word_-_14th_june_2013. Checklist adding reporting in acupuncture studies as suggestedby the STRICTA criteria.
(DOCX)

S3 File AcuTA_Trial Protocol_1.0. Study protocol as approved by the Ethics Committee.
(PDF)

## References

[1] Isserstedt W, Middendorff E, Kandulla M, Borchert L, Leszczensky M. The Economic and Social Conditions of Student Life in the Federal Republic of Germany in 2009 [Die wirtschaftliche und soziale Lage der Studierenden in der Bundesrepublik Deutschland 2006] - 19th Social Survey of the Deutsche Studentenwerk conducted by HIS Hochschul-Informations-System Federal Ministry of Education and Research (BMBF); 2010 [March 20th 2013]. Available from: http://www.sozialerhebung.de/download/19/Soz19_KurzfassungEnglisch_Internet.pdf.

[2] Grüner F. Dissertation: Lernstrategien und Prüfungsangst bei Studierenden der Studiengänge Humanmedizin und Lehramt: Würzburg; 2010.

[3] Mandl H, Pekrun R, Götz T. Handbuch Lernstrategien. Emotionsregulation: Vom Umgang mit Prüfungsangst.: Hogrefe Verlag GmbH & Co, Göttingen; 2006.

[4] Middendorff E, Poskowsky J, Isserstedt W. Formen der Stresskompensation und Leistungssteigerung bei Studierenden: HISBUS-Befragung zur Verbreitung und zu Mustern von Hirndoping und Medikamentenmissbrauch. German Health Ministery [Bundesministerium für Gesundheit], 2012.

[5] Diekelmann S, Landolt HP, Lahl O, Born J, Wagner U. Sleep loss produces false memories. PloS one. 2008;3(10):e3512 10.1371/journal.pone.0003512 ; PubMed Central PMCID: PMC2567433. 18946511 PMC2567433

[6] Greely H, Sahakian B, Harris J, Kessler RC, Gazzaniga M, Campbell P, et al Towards responsible use of cognitive-enhancing drugs by the healthy. Nature. 2008;456(7223):702–5. 10.1038/456702a . 19060880

[7] Shu S, Li TM, Fang FF, He HL, Zhou QH, Gu W, et al [Relieving pre-exam anxiety syndrome with wrist-ankle acupuncture: a randomized controlled trial]. Zhong xi yi jie he xue bao = Journal of Chinese integrative medicine. 2011;9(6):605–10. . 21669163 10.3736/jcim20110605

[8] Isoyama D, Cordts EB, de Souza van Niewegen AM, de Almeida Pereira de Carvalho W, Matsumura ST, Barbosa CP. Effect of acupuncture on symptoms of anxiety in women undergoing in vitro fertilisation: a prospective randomised controlled study. Acupuncture in medicine: journal of the British Medical Acupuncture Society. 2012;30(2):85–8. 10.1136/acupmed-2011-010064 . 22499825

[9] Zhao Z, Kim SC, Zhao R, Wu Y, Zhang J, Liu H, et al The tegmental-accumbal dopaminergic system mediates the anxiolytic effect of acupuncture during ethanol withdrawal. Neuroscience letters. 2015;597:143–8. 10.1016/j.neulet.2015.04.045 . 25936591

[10] Kim DH, Kim NJ, Zhao RJ, Kim DH, Yang CH, Kim HY, et al Effects of acupuncture on the anxiety-like behavior induced by withdrawal from chronic morphine use. Neuroscience letters. 2018;664:38–42. 10.1016/j.neulet.2017.11.008 . 29126776

[11] Chan J, Briscomb D, Waterhouse E, Cannaby AM. An uncontrolled pilot study of HT7 for 'stress'. Acupuncture in medicine: journal of the British Medical Acupuncture Society. 2002;20(2–3):74–7. . 12216604 10.1136/aim.20.2-3.74

[12] Sharifi Rizi M, Shamsalinia A, Ghaffari F, Keyhanian S, Naderi Nabi B. The effect of acupressure on pain, anxiety, and the physiological indexes of patients with cancer undergoing bone marrow biopsy. Complementary therapies in clinical practice. 2017;29:136–41. Epub 2017/11/11. 10.1016/j.ctcp.2017.09.002 . 29122251

[13] Kirschbaum C, Pirke KM, Hellhammer DH. The 'Trier Social Stress Test'—a tool for investigating psychobiological stress responses in a laboratory setting. Neuropsychobiology. 1993;28(1–2):76–81. doi: 119004. 10.1159/000119004 . 8255414

[14] Irnich D, Salih N, Offenbacher M, Fleckenstein J. Is sham laser a valid control for acupuncture trials? Evidence-based complementary and alternative medicine: eCAM. 2011;2011:485945 10.1093/ecam/neq009 PubMed PMID: 21772922; PubMed Central PMCID: PMC3135659. 21772922 PMC3135659

[15] Hinz A, Daig I, Petrowski K, Brahler E. [Mood in the German population: norms of the Multidimensional Mood Questionnaire MDBF]. Psychotherapie, Psychosomatik, medizinische Psychologie. 2012;62(2):52–7. Epub 2012/01/25. 10.1055/s-0031-1297960 . 22271232

[16] Gaab J, Rohleder N, Nater UM, Ehlert U. Psychological determinants of the cortisol stress response: the role of anticipatory cognitive appraisal. Psychoneuroendocrinology. 2005;30(6):599–610. 10.1016/j.psyneuen.2005.02.001 15808930

[17] Spielberger CD. Manual for state-trait anxiety inventory (STAI: Form Y) Palo Alto, CA: Consulting Psychologist Press; 1983.

[18] Vincent C, Lewith G. Placebo controls for acupuncture studies. J R Soc Med. 1995;88(4):199–202. ; PubMed Central PMCID: PMCPMC1295163. 7745565 PMC1295163

[19] Kamody RC, Thurston IB, Decker KM, Kaufman CC, Sonneville KR, Richmond TK. Relating shape/weight based self-esteem, depression, and anxiety with weight and perceived physical health among young adults. Body image. 2018;25:168–76. Epub 2018/04/21. 10.1016/j.bodyim.2018.04.003 . 29677688

[20] Henze GI, Zankert S, Urschler DF, Hiltl TJ, Kudielka BM, Pruessner JC, et al Testing the ecological validity of the Trier Social Stress Test: Association with real-life exam stress. Psychoneuroendocrinology. 2017;75:52–5. Epub 2016/10/25. 10.1016/j.psyneuen.2016.10.002 . 27771565

[21] MacPherson H, Vertosick E, Lewith G, Linde K, Sherman KJ, Witt CM, et al Influence of control group on effect size in trials of acupuncture for chronic pain: a secondary analysis of an individual patient data meta-analysis. PloS one. 2014;9(4):e93739 Epub 2014/04/08. 10.1371/journal.pone.0093739 ; PubMed Central PMCID: PMCPMC3976298. 24705624 PMC3976298

[22] MacPherson H, White A, Cummings M, Jobst K, Rose K, Niemtzow R, et al Standards for reporting interventions in controlled trials of acupuncture: The STRICTA recommendations. STandards for Reporting Interventions in Controlled Trails of Acupuncture. Acupuncture in medicine: journal of the British Medical Acupuncture Society. 2002;20(1):22–5. . 11926601 10.1136/aim.20.1.22

[23] Reschke-Hernandez AE, Okerstrom KL, Bowles Edwards A, Tranel D. Sex and stress: Men and women show different cortisol responses to psychological stress induced by the Trier social stress test and the Iowa singing social stress test. Journal of neuroscience research. 2017;95(1–2):106–14. Epub 2016/11/22. 10.1002/jnr.23851 ; PubMed Central PMCID: PMCPMC5120613. 27870432 PMC5120613

[24] Nedeljkovic M, Ausfeld-Hafter B, Streitberger K, Seiler R, Wirtz PH. Taiji practice attenuates psychobiological stress reactivity—a randomized controlled trial in healthy subjects. Psychoneuroendocrinology. 2012;37(8):1171–80. 10.1016/j.psyneuen.2011.12.007 . 22222120

[25] Schweden TL, Pittig A, Brauer D, Klumbies E, Kirschbaum C, Hoyer J. Reduction of depersonalization during social stress through cognitive therapy for social anxiety disorder: A randomized controlled trial. Journal of anxiety disorders. 2016;43:99–105. Epub 2016/09/21. 10.1016/j.janxdis.2016.09.005 . 27648752

[26] Wichmann S, Kirschbaum C, Lorenz T, Petrowski K. Effects of the cortisol stress response on the psychotherapy outcome of panic disorder patients. Psychoneuroendocrinology. 2016;77:9–17. Epub 2016/12/18. 10.1016/j.psyneuen.2016.11.030 . 27987430

[27] Haker E, Egekvist H, Bjerring P. Effect of sensory stimulation (acupuncture) on sympathetic and parasympathetic activities in healthy subjects. Journal of the Autonomic Nervous System. 2000;79(1):52–9. 10.1016/S0165-1838(99)00090-9 10683506

[28] Schneider A, Weiland C, Enck P, Joos S, Streitberger K, Maser-Gluth C, et al Neuroendocrinological effects of acupuncture treatment in patients with irritable bowel syndrome. Complementary therapies in medicine. 2007;15(4):255–63. 10.1016/j.ctim.2006.12.002 18054727

[29] Klausenitz C, Hacker H, Hesse T, Kohlmann T, Endlich K, Hahnenkamp K, et al Auricular Acupuncture for Exam Anxiety in Medical Students-A Randomized Crossover Investigation. PloS one. 2016;11(12):e0168338 Epub 2016/12/30. 10.1371/journal.pone.0168338 . 28033320 PMC5198977

[30] de Lorent L, Agorastos A, Yassouridis A, Kellner M, Muhtz C. Auricular Acupuncture Versus Progressive Muscle Relaxation in Patients with Anxiety Disorders or Major Depressive Disorder: A Prospective Parallel Group Clinical Trial. Journal of acupuncture and meridian studies. 2016;9(4):191–9. Epub 2016/08/25. 10.1016/j.jams.2016.03.008 . 27555224

[31] Goyata SL, Avelino CC, Santos SV, Souza Junior DI, Gurgel MD, Terra Fde S. Effects from acupuncture in treating anxiety: integrative review. Revista brasileira de enfermagem. 2016;69(3):602–9. Epub 2016/06/30. 10.1590/0034-7167.2016690325i . 27355312

[32] So EW, Ng EH, Wong YY, Yeung WS, Ho PC. Acupuncture for frozen-thawed embryo transfer cycles: a double-blind randomized controlled trial. Reproductive biomedicine online. 2010;20(6):814–21. Epub 2010/04/13. 10.1016/j.rbmo.2010.02.024 . 20382081

[33] Kwong EY, Yiu EM. A preliminary study of the effect of acupuncture on emotional stress in female dysphonic speakers. Journal of voice: official journal of the Voice Foundation. 2010;24(6):719–23. Epub 2010/01/20. 10.1016/j.jvoice.2009.05.005 . 20083382

[34] Akimoto T, Nakahori C, Aizawa K, Kimura F, Fukubayashi T, Kono I. Acupuncture and responses of immunologic and endocrine markers during competition. Medicine and science in sports and exercise. 2003;35(8):1296–302. Epub 2003/08/06. 10.1249/01.MSS.0000078934.07213.25 . 12900681

[35] Villas-Boas JD, Dias DP, Trigo PI, Almeida NA, de Almeida FQ, de Medeiros MA. Acupuncture Affects Autonomic and Endocrine but Not Behavioural Responses Induced by Startle in Horses. Evidence-based complementary and alternative medicine: eCAM. 2015;2015:219579 Epub 2015/09/29. 10.1155/2015/219579 ; PubMed Central PMCID: PMCPMC4568046. 26413116 PMC4568046

[36] Kim SA, Lee BH, Bae JH, Kim KJ, Steffensen SC, Ryu YH, et al Peripheral afferent mechanisms underlying acupuncture inhibition of cocaine behavioral effects in rats. PloS one. 2013;8(11):e81018 Epub 2013/11/22. 10.1371/journal.pone.0081018 ; PubMed Central PMCID: PMCPMC3832370. 24260531 PMC3832370

[37] Zhao RJ, Yoon SS, Lee BH, Kwon YK, Kim KJ, Shim I, et al Acupuncture normalizes the release of accumbal dopamine during the withdrawal period and after the ethanol challenge in chronic ethanol-treated rats. Neuroscience letters. 2006;395(1):28–32. 10.1016/j.neulet.2005.10.043 16289320

[38] Zhao Z, Kim SC, Zhao R, Wu Y, Zhang J, Liu H, et al The tegmental–accumbal dopaminergic system mediates the anxiolytic effect of acupuncture during ethanol withdrawal. Neuroscience letters. 2015;597:143–8. 10.1016/j.neulet.2015.04.045 25936591

[39] Park HJ, Park HJ, Chae Y, Kim JW, Lee H, Chung JH. Effect of acupuncture on hypothalamic-pituitary-adrenal system in maternal separation rats. Cellular and molecular neurobiology. 2011;31(8):1123–7. Epub 2011/06/07. 10.1007/s10571-011-9718-x . 21643998 PMC11498392

