# Supplementary material for: Effects of single-point acupuncture (HT7) in the prevention of test anxiety: Results of a RCT
Source: PLoS One. 2018 Aug 30;13(8):e0202659. doi: 10.1371/journal.pone.0202659 (PMC6116988; doi:10.1371/journal.pone.0202659)
Supplement: S3 File — Study protocol as approved by the Ethics Committee. (PDF) [file pone.0202659.s003.pdf]

Clinical Trial Protocol

Acupuncture randomized trial for preventing test anxiety

Version 1.0

Short Title: **AcuTA**: Acupuncture in Test Anxiety

Sponsor: University of Regensburg,
presented by: PD Dr. Karl-Peter Ittner

Principle Investigator: Dr. Johannes Fleckenstein

Lecturer for Acupuncture
University of Regensburg

Department of Traditional Chinese Medicine and Acupuncture
Institute of Complementary Medicine KIKOM
University of Bern, Inselspital
CH-3010 Bern, Switzerland

Co-Investigator: PD Dr. Karl-Peter Ittner

Department of Anaesthesiology
University of Regensburg
Franz-Joseph-Strauß Allee 11
93053 Regensburg

Content

1. Investigator 3
2. Background 3
3. Objectives and purpose 5
4. Trial design 6
5. Selection of the trial subjects 8
6. Assessment of effectiveness 9
7. Assessment of safety 10
8. Sample Size Estimation and Statistics 11
9. Trial-specific preventive measures and duties 12
11. Ethical considerations 13
12. Quality control and quality assurance: description of measures 13
Protocol Approval Signatures 16
13. References 17

1. Investigator

Dr. Johannes Fleckenstein is head of the Department of Traditional Chinese Medicine and Acupuncture, Institute of Complementary Medicine KIKOM, University of Bern since August 2012 and a scientist registrar for many years at the Multidisciplinary Pain Centre, Department of Anaesthesiology, University of Munich, experienced in the management of clinical trials. He is a lecturer for acupuncture at the University of Regensburg since 2011. He is experienced in the conception and enrolment of multiple clinical trials. He is an approved Principle Investigator for Clinical Trials (GCP-ICH), University of Erlangen.

2. Background

Test anxiety is a well-known phenomenon in general population, but only few scientific advances have been made in order to fully understand and prevent this circumstance.

To our knowledge, there is only poor epidemiological data on the prevalence of test anxiety in the general population. There is some knowledge about test anxiety in German students. The German Federal Ministry of Education and Research reports, that 13% of all first year undergraduate students accept advising services regarding test anxiety. More female students (16% of all female students which study for the first time) seek advice in this topic than their male fellow students (10% of all male students which study for the first time) (Isserstedt et al., 2010). Test anxiety seems to be more prevalent in students, which dropped their first choice of study and start another. In this group 17% accepted advising services. It could also be shown, that the amount of students which are affected with test anxiety and need help rises with the age of the students (Isserstedt et al., 2010).

Anxiety in combination with tests has different components. There is an affective component, which appears in an unpleasurable, nervous feeling of affective excitement, a cognitive compound, which contains concerns for an impending failure and its possible consequences, a physiological component, for example an increased heart rate, sweating or nausea, and a motivational component, which means escape and avoidance tendencies (Grüner, 2010). The affective and the physiological component are often designated as “emotionality”, the cognitive component as “worry” (Mandl et al., 2006). Important is also the distinction between currently experienced test anxiety, the so called state test anxiety on the one hand and habitual personality-specific test anxiety on the other hand, which is called trait test anxiety (Mandl et al., 2006).There are two main

areas of fear genesis. One is subjective lack of control, which leads to failure expectancy and uncertainity. The second is failure and its consequences (Mandl et al., 2006).

Various effects, possibly related to test anxiety, have been described. First, suffering test anxiety, the attention declines. If someone is afraid of a test his/her thoughts only deal with the possibility of failure and its consequences. Second, students attention for the preparation of the test is reduced. Accordingly the performance in complex, difficult tasks, which require attention, decreases. The effect on motivation is ambivalent. Test anxiety is known to reduce interest and motivation but can be beneficial too, as students are more focused on avoiding errors. In regard to task condition and individual motivation, test anxiety may reduce or increase students' performance, but in general it is supposed to weakening abilities to solving cognitive challenging tasks (Mandl et al., 2006).

Middendorf et al. showed in their survey that the most popular options for students to relief anxiety and stress is meeting with friends (69%) and entertainment (67%). Other remedies are sleeping (63%), sport activities (58%) or relaxing and wellness (46%) (Middendorff et al., 2012). Drinking coffee is a common strategy for staying awake and concentrated. Diekelmann et al. wanted to know, if caffeine has an effect on memory after sleep deprivation. The group given caffeine had 10% less false memories than those who did not receive any (Diekelmann et al., 2008).

Bernstein et al. tested the effect of caffeine on students. In a small sample size, there was indication that caffeine enhanced performance on a test of attention and on a motor task. Children also reported feeling less "sluggish" but somewhat more anxious (Bernstein et al., 1994). Energy drinks are also used for more alertness and attention. They usually contain high concentrations of glucose and caffeine and shall lead to an even higher increase of memory performance than single ingredients by its own (Forstl, 2009)

The number of students which use neuro enhancement to improve their performance and to prevent test anxiety, is increasing. A US-survey estimated that almost 7% of students in US universities have used prescription stimulants against anxiety, and that on some campuses, up to 25% of students had used them in the past year (Greely et al., 2008)

Middendorf et al. found out, that 12% of all questioned students in 2012 had used one or more substances to cope with the requirements of their study since they started their studies. Five percent of these students take drugs which are only available on prescription, analgetics, sedatives, psychostimulants or stimulants, another 5% are so called soft-enhancers. This group tries to optimize their performance with vitamins, homeopathic or herbal remedies or coffein (Middendorff et al., 2012). Performance-

enhancing drugs are mostly used in exam preparation (enhancers 55%, soft-enhancers 58%), general stress (enhancers 53%, soft-enhancers 35%) and testing situations (enhancers 45%, soft-enhancers 60%).

Greely et al. found out that the most popular drugs used for cognitive enhancement at present are the stimulantsmethyphenidate and mixed amphetamine salts,both of them normally prescribed for the treatment of attention deficit hyperactivity disorder (ADHD). A newer drug, modafinil is approved for the treatment of fatigue caused by narcolepsy, sleep apnoea and shift-work sleep disorder and has been reported to be beneficial in anxiety (Greely et al., 2008).

3. Objectives and purpose

3.1 Scientific Evidence

Shu et al. could show that Wrist-Ankle-Acupuncture can relieve symptoms of pre-exam anxiety syndrome significantly and that this therapy is highly safe (Shu et al., 2011). In several trials was explored if acupuncture has an effect in dealing with anxiety. Isoyama et al. inserted in the test group needles an points HT7, PC6, CV17, GV20 and Yintang. In the control group needles were inserted in areas near but not corresponding to acupuncture points. The results indicate that acupuncture can reduce anxiety symptoms observed by the reduction of psychological parameters of women undergoing IVF (Isoyama et al., 2012). Another study demonstrated that bilateral acupuncture needling at HT7 was an effective method for reducing the rating of “psychological stress” in 16 out of a group of 17 volunteers, recruited from staff in a hospice. Ratings were made in using Edinburgh Postnatal Depression Scale (EPDS). Four brief acupuncture sessions were performed at weekly intervals. The greatest fall in the EPDS scores was observed within the first two treatments. At the end of the study, there was an average reduction of 44% in the EPDS scores (Chan et al., 2002). Another single-blind randomized controlled trial was made to test the effectiveness of auriculotherapy in the reduction of anxiety levels in Portuguese nursing school students. The Trait-Anxiety Inventory State was applied at the beginning of the study, after 8 and 12 sessions and at follow-up (15 days). They showed statistically significant differences post hoc between the control and auriculotherapy groups. Auriculotherapy with Shenmen and Brain Stem points was more effective than sham points for reduction of anxiety levels in Nursing students (do Prado et al., 2012).

Finally, trials have shown that single-point acupuncture is strong enough to cause relevant clinical effects (Fleckenstein et al., 2009; Lee and Fan, 2009). This is of important practicability in acute states, such as pain or emesis. The administration of a single needle at acupuncture points guaranteeing specific effects, is not only satisfying for the patient - receiving immediate relief - but also the acupuncturist - giving rapid help. Single point acupuncture can be provided quickly in indicated disorders.

Altogether, the investigation of single point effects in test anxiety could be of general interest. The acupuncture point with the most convincing evidence up to date is Heart 7. Yet, its effectiveness has mainly been chosen in combination with other acupuncture points and not as single remedy in test anxiety. Therefore we establish a trial investigating the immediate needling effects at Heart 7 on the reduction of test anxiety.

3.2 Justified choice of a trial subject population

As described above, students figure a population especially burdened by test anxiety. Providing them other than neuro-enhancing drugs might reduce anxiety and improve cognitive function. In addition, students are continuously exposed to exam situations, so it will be eased to recruit sufficient participants.

3.3 Hypothesis that could be established on the basis of the issues studied.

A bilateral single needle acupuncture treatment at the acupuncture point Heart 7 immediately reduces test anxiety.

4. Trial design

4.1. Primary end point

The primary end point is the process of the salivary cortisol reaction initiated by a TSST when comparing acupuncture at Heart 7 with laser acupuncture treatment.

4.2 Trial design:

We conduct a randomized controlled, two-armed pilot trial to investigate the effectiveness of a single acupuncture treatment at bilateral acupuncture point Heart 7 when compared to no acupunctural treatment of acute test anxiety. Test anxiety will be induced using the

protocol of the Trier Social Stress Test, a validated instrument for provocation of psychobiological stress (Kirschbaum et al., 1993).

Participants will be allocated to one of two trial-arms receiving either single needle acupuncture at Heart 7 (ACU) or laser acupuncture (LAS).

Acupuncture Needles (Seirin® 0.15 mm diameter and 15 mm length) are placed bilaterally into the acupuncture point Heart 7 (ACU). Deqi has to be achieved. Needle-in time is 20 minutes. Participants will lay comfortably on a lounger.

Laser Acupuncture will be performed at the same acupuncture point, bilaterally, without palpating or touching the skin (laser pen manufactured by 3B Scientific,GmbH, Hamburg, Germany). Treatment is one minute per point with additional 18 minutes of resting time.

The experimental sessions are conducted between 1300 h and 1800 h. The timing of the stress test performance is balanced between participants in the two study groups. Participants are told to refrain from eating and drinking anything but water for 2 h and from intense physical activity, caffeine, nicotine, and alcohol during the 24 h before the experiment. The ECG recording equipment is attached first and the recording is started. We use the Trier Social Stress Test (TSST) combining a 10 min preparation phase followed by a 5 min mock job interview, and a 5 min mental arithmetic exercise (Kirschbaum et al., 1993). Both tasks are performed 2 m in front of two evaluative panel members dressed in white laboratory coats, and a conspicuous video camera and microphone. The socio-evaluative character of this performance task is further underlined by presenting the panel members (a retired male finance manager and a female psychologist) as experts in evaluation of nonverbal behaviour. The TSST reliably activates HPA-axis and the sympathetic nervous system. During recovery, subjects remained seated in a quiet room for 60 min.

Timeflow of the trial:

Day	Event	Timepoint
**Recruitment**	▪ Informed Consent<br>▪ Inclusion<br>▪ Appointment TSST	1-2 weeks before TSST
**Trier Social Stress Test**	▪ Check-In<br>▪ Confirmation Consent<br>▪ Inclusion Criteria	-60 min

	▪ Resting Time 20 minutes	
	▪ Saliva samples	-40min
	▪ Questionnaires	
	▪ Intervention	-30 min
	▪ (Needle- or Laseracupuncture)	(incl. 18 min Resting Time)
	▪ Saliva samples	-10 min
	▪ Questionnaires	
	▪ Resting Time	-10 min
	▪ **TSST: Anticipation**	**0 min**
	▪ **TSST: Talk/Arithmetic**	**10 min**
	▪ Saliva samples	10 min
	▪ Questionnaires	20 min
	▪ Resting Time	20 min
	▪ Saliva samples	30 min
	▪ Questionnaires	45 min
		60 min
**End of the trial**		60 min

4.3. Randomisation

The randomisation procedure will be performed by the Department of Biometry. They prepare on the basis of a computer generated allocation list a series of sealed envelopes which are sequentially numbered from 1 to 24, taken possible participant losses into account, this allocating the patients to two trial groups. The allocation ratio between groups is 1:1.

ACU: single acupuncture at point Heart 7

LAS: Laser acupuncture control

5. Selection of the trial subjects

5.1. Recruitment

Possible participants are male students of the Medical School at the University of Regensburg. The trial will be announced orally, sharing hand-outs and by e-mail. All interested students will be informed of nature, importance, treatment methods, risks and consequences of the trial. All students willing to participate will be listed. Inclusion into

the trial starts the day of the oral examination. Respective students will be assessed for their test anxiety on a visual analogue scale ranging from 0 to 10 (with ten being maximum anxious). Students achieving a score above 4 cm VAS and the below mentioned inclusion and exclusion criteria will be included into the trial, if written consent is signed. It is intended that all included students will receive a cinema-voucher for their participation.

5.2. Inclusion criteria:

- Test Anxiety in the clinical history
- Male medical students 3rd to 5th year
- Compliance
- Age > 18 years
- Smoking cessation for 24 hours

5.3. Exclusion criteria:

- Severe physical or psychical illness
- Psychiatric record in medical history
- Continuous uptake of antipsychiatric medication, tranquilizers or neuro-enhancers
- Acupuncture treatment within the last 4 weeks
- Hang-over
- Drug consumption
- Smoking (> 5 cigarettes/ day)

6. Assessment of effectiveness

6.1. Effectiveness parameters:

- Anxiety Questionnaires will be assessed at baseline, after intervention, and at the end of the TSST
    - Test Anxiety on a Visual Analogue Scale (ranging 0 to 10, with 10 being most anxious)

- Primary Appraisal Secondary Appraisal (PASA) Questionnaire
- Multidimensional Mood State Questionnaire (MDBF)

- Physiologic parameters will be assessed at baseline, after intervention, and at the end of the TSST
    - Heart Rate Variability
    - Heart Rate
    - Physical activity
- Saliva samples will be obtained at 10-to 30-min intervals for subsequent analysis of cortisol and α-amylase in saliva.

7. Assessment of safety

7.1. Safety parameters:

- Side effects

7.2. Adverse Reactions

An Adverse Event/Experience (AE) is any untoward medical occurrence in a patient or in a clinical investigation subject administered a pharmaceutical product and which does not necessarily have a causal relationship with this treatment. Any AE has to be documented in the CRF on the respective Adverse Event Report Form according to the GCP-ICH guidelines

An AE can therefore be any unfavourable and unintended sign (including an abnormal laboratory finding), symptom or disease temporally associated with the use of a medicinal product, whether or not related to the treatment. All noxious and unintended responses to a medicinal product should be considered adverse drug reactions. The phrase responses to a medicinal product means that a causal relationship between a medicinal product and an AE is at least a reasonable possibility, i.e. the relationship cannot be ruled out.

The relationship of AEs to the investigational products being studied should be determined as follows:

Definitely Related: When a temporal relationship of the onset of the event, relative to the administration of the drug is reasonable and there is no other cause to explain the event or a re-challenge is positive.

Probably Related: When a temporal relationship of the onset of the event, relative to the administration of the drug is reasonable and the event is more likely to be explained by the medicinal product than by another cause.

Possibly Related: When a temporal relationship of the onset of the event, relative to the administration of the drug is reasonable, but the event could have been due to an equally likely cause.

Unlikely: When a temporal relationship of the onset of the event, relative to the administration of the drug is unlikely.

Not Related: When a temporal relationship of the onset of the event, relative to the administration of the drug is not reasonable or when another cause can explain the occurrence of the event by itself.

Both principle investigators are anaesthesiologists and skilled in emergency medicine. The trial takes place at the University Hospital of the University of Regensburg. In case of a Suspected Unexpected Serious Adverse Reaction (SUSAR) additional assistance by the hospital's emergency team is available and secondary attendance guaranteed.

8. Sample Size Estimation and Statistics

8.1 A power analysis was performed using G*Power (University of Düsseldorf, Germany) estimating a small to medium effect size (d = 0.29) and a time-dependent progression (estimated 5 timepoints), this suggesting a total sample size of 24 subjects.

F tests - ANOVA: Repeated measures, within-between interaction

Analysis: A priori: Compute required sample size

**Input:**	Effect size f	=	0.29
	α err prob	=	0.05
	Power (1-β err prob)	=	0.95
	Number of groups	=	2
	Number of measurements	=	5
	Corr among rep measures	=	0.5
	Nonsphericity correction ε	=	1
**Output:**	Noncentrality parameter λ	=	20.1840000
	Critical F	=	2.4752774
	Numerator df	=	4.0000000

Denominator df	=	88.0000000
Total sample size	=	24
Actual power	=	0.9559026

8.2 The primary endpoint is the difference in saliva cortisol between both groups. Analysis of the main outcome measure will be performed using a repeated measures ANOVA. In case that data is not normally distributed equivalent non-parametric tests will be performed. Other data will be analysed in this regard, too.

9. Trial-specific preventive measures and duties

9.1 Responsibilities of the Sponsor

The sponsor is only responsible for obtaining the approval from the respective main research ethics committee ("federführende Ethikkommision") before initiation of the trial.

The sponsor announces a Leader of the clinical trial (LKP) who has more than two years of experience in the field of clinical trial and holds a medical license.

9.2 Duties of the investigator

By signing this protocol the local investigator declares his/her commitment:

- to not enrol any person dependent on him/her or the sponsor based on the principles of ICH-GCP
- to follow the regulations for data security as given by the respective main research ethics committee
- to inform the subjects of the transmission of their pseudonymized data based on documentation and transmission obligations (§ 12 and § 13 GCP-V) and to make sure that subjects unwilling to give consent to the processing of their data are not included into the trial
- to certify that he/she was informed of the risks of the clinical trial in a way based on § 40 Abs. 1, Satz 3 Nr. 7 AMG
- to be qualified by education, training and experience to assume responsibility for the proper conduct of the subject
- to be thoroughly familiar with the appropriate use of interventions, as described in the protocol, the product information and other information sources provided by the sponsor
- to be aware of GCP and the applicable regulatory requirements

- to maintain a list of appropriately qualified persons to whom the investigator has delegated significant subject related duties (if applicable).

11. Ethical considerations

11.1 Ethics Committee

In addition to protocol and amendments the subject information and informed consent, and any other written information to be provided to the trial subjects have to be approved by the respective research ethics committee (EC, “federführende Ethikkommision”). Any substantial amendments to the protocol or subsequent changes to the informed consent form as a result of changes to the protocol must also be sent to the EC. Records of the EC review and opinion of all documents pertaining to this trial must be kept on file by the investigator and are subject to regulatory authority and / or sponsor inspection during or after completion of the trial.

The sponsor will provide a safety update of the trial to the EC, including line listing, individual reports of SUSARs, if applicable, annually or more frequently if requested.

11.2 Risk assessment.

The recent safety report on acupuncture is based on 2.2 million acupuncture sessions (Witt et al., 2009). Nine percent of patients reported experiencing at least 1 adverse effect. Adverse effects requiring treatment occurred in 2.2% of patients. Overall, adverse effects were reported in 1.1%. Common adverse effects were minor bleeding or haematoma (6.1%) and pain (1.7%) followed by vegetative symptoms (0.7%). Bleeding or haematoma was the predominant adverse effect (58% of all adverse effects. Adverse effects which indicate negligence or malpractice (i.e. broken or forgotten needle, pneumothorax, burns after moxibustion) occurred in 0.1% of all adverse effects. There from we conclude acupuncture comprising triggerpoint needling techniques being a low risk treatment.

12. Quality control and quality assurance: description of measures

12.1 Quality Assessment

Protocol violations are major deviations from the procedures outlined in this document like:

- Missed evaluations
- Incorrect timing of evaluations

- Non-compliance with investigational medicinal product, if applicable
- Self-medication beyond the protocol
- Any non-adherence to the protocol that would have an impact to the subject's rights, safety or welfare.

After a subject has been enrolled, it is the investigator's responsibility to make a reasonable effort to correct any protocol violations and to continue the subject's participation in the trial, if possible.

Protocol violations do not constitute a justification for withdrawal of a subject from the trial themselves.

Protocol violations will be reported to the sponsor/sponsor delegated person during the course of the trial in the monitoring reports.

All protocol violations will be listed and the impact on the evaluation of the subjects concerned will be discussed prior to statistical analysis.

12.2 Archiving

The sponsor must retain all essential documents for the duration of at least 10 years after end or stop of trial. The sponsor must archive all trial related documents according to regulatory requirements.

The investigator should maintain all subject documents after completion of the clinical trial so that they will be available for inspections by the authorities. The investigator will be responsible for the storage.

The following retention periods will apply after completion or stop of the clinical trial:

- All essential documents and trial related data must be retained securely for at least 10 years,
- Medical records and other source documents for the longest possible period allowed by the hospital, the institution or the private practice.

The investigator/institution should take arrangements to prevent accidental or premature destruction and illegitimate access to these documents.

12.3 Clinical Record Files (CRF)

The investigator will record the participation in the trial, the frequency of the trial visits, the relevant medical data, the concomitant treatment and the occurrence of adverse events in the CRF of each subject. The investigator has ultimate responsibility for the accuracy, authenticity, timely collection and reporting of all clinical, safety, laboratory data entered on the CRFs. All these data may only be entered into the CRF by authorized trial personnel as promptly as possible.

Protocol Approval Signatures

Principle Investigator:

Dr. med. Johannes Fleckenstein

Date ____________ Signature ____________

Co- Investigator:

PD Dr. med Karl-Peter Ittner

Date ____________ Signature ____________

13. References

Bernstein, G.A., Carroll, M.E., Crosby, R.D., Perwien, A.R., Go, F.S., Benowitz, N.L., 1994. Caffeine effects on learning, performance, and anxiety in normal school-age children. Journal of the American Academy of Child and Adolescent Psychiatry 33, 407-415.

Chan, J., Briscomb, D., Waterhouse, E., Cannaby, A.M., 2002. An uncontrolled pilot study of HT7 for 'stress'. Acupuncture in medicine : journal of the British Medical Acupuncture Society 20, 74-77.

Diekelmann, S., Landolt, H.P., Lahl, O., Born, J., Wagner, U., 2008. Sleep loss produces false memories. PloS one 3, e3512.

do Prado, J.M., Kurebayashi, L.F., da Silva, M.J., 2012. [Auriculotherapy effectiveness in the reduction of anxiety in nursing students]. Revista da Escola de Enfermagem da U S P 46, 1200-1206.

Fleckenstein, J., Lill, C., Ludtke, R., Gleditsch, J., Rasp, G., Irnich, D., 2009. A single point acupuncture treatment at large intestine meridian: a randomized controlled trial in acute tonsillitis and pharyngitis. The Clinical journal of pain 25, 624-631.

Forstl, H., 2009. [Neuro-enhancement. Brain doping]. Der Nervenarzt 80, 840-846.

Greely, H., Sahakian, B., Harris, J., Kessler, R.C., Gazzaniga, M., Campbell, P., Farah, M.J., 2008. Towards responsible use of cognitive-enhancing drugs by the healthy. Nature 456, 702-705.

Grüner, F., 2010. Dissertation: Lernstrategien und Prüfungsangst bei Studierenden der Studiengänge Humanmedizin und Lehramt. Würzburg.

Isoyama, D., Cordts, E.B., de Souza van Niewegen, A.M., de Almeida Pereira de Carvalho, W., Matsumura, S.T., Barbosa, C.P., 2012. Effect of acupuncture on symptoms of anxiety in women undergoing in vitro fertilisation: a prospective randomised controlled study. Acupuncture in medicine : journal of the British Medical Acupuncture Society 30, 85-88.

Isserstedt, W., Middendorff, E., Kandulla, M., Borchert, L., Leszczensky, M., 2010. The Economic and Social Conditions of Student Life in the Federal Republic of Germany in 2009 [Die wirtschaftliche und soziale Lage der Studierenden in der Bundesrepublik Deutschland 2006] - 19th Social Survey of the Deutsche Studentenwerk conducted by HIS Hochschul-Informations-System Federal Ministry of Education and Research (BMBF).

Kirschbaum, C., Pirke, K.M., Hellhammer, D.H., 1993. The 'Trier Social Stress Test'--a tool for investigating psychobiological stress responses in a laboratory setting. Neuropsychobiology 28, 76-81.

Lee, A., Fan, L.T., 2009. Stimulation of the wrist acupuncture point P6 for preventing postoperative nausea and vomiting. Cochrane Database Syst Rev, CD003281.

Mandl, H., Pekrun, R., Götz, T., 2006. Handbuch Lernstrategien. Emotionsregulation: Vom Umgang mit Prüfungsangst. Hogrefe Verlag GmbH & Co, Göttingen.

Middendorff, E., Poskowsky, J., Isserstedt, W., 2012. Formen der Stresskompensation und Leistungssteigerung bei Studierenden: HISBUS-Befragung zur Verbreitung und zu Mustern von Hirndoping und Medikamentenmissbrauch. German Health Ministery [Bundesministerium für Gesundheit].

Shu, S., Li, T.M., Fang, F.F., He, H.L., Zhou, Q.H., Gu, W., Zhou, S., 2011. [Relieving pre-exam anxiety syndrome with wrist-ankle acupuncture: a randomized controlled trial]. Zhong xi yi jie he xue bao = Journal of Chinese integrative medicine 9, 605-610.
